# Minimally Invasive Modified Nuss Procedure for Repair of Pectus Excavatum in Pediatric Patients: Single-Centre Retrospective Observational Study

**DOI:** 10.3390/children8111071

**Published:** 2021-11-20

**Authors:** Miro Jukić, Ivan Mustapić, Tomislav Šušnjar, Zenon Pogorelić

**Affiliations:** 1 Department of Pediatric Surgery, University Hospital of Split, Spinčićeva 1, 21 000 Split, Croatia; tomislav.susnjar@optinet.hr (T.Š.); zpogorelic@gmail.com (Z.P.); 2 Department of Surgery, School of Medicine, University of Split, Šoltanska 2, 21 000 Split, Croatia; ivan.musta@gmail.com

**Keywords:** pectus excavatum, funnel chest, adolescents, Nuss procedure, minimally invasive surgery

## Abstract

Background: The treatment of pectus excavatum can be conservative or surgical. The aim of this study was to determine the factors influencing the outcomes of treatment after a minimally invasive Nuss corrective surgery procedure in pediatric patients. Methods: A total of 30 patient who underwent a minimally invasive Nuss corrective procedure for pectus excavatum from 1 January 2014 to 31 December 2020 were included in thisretrospective study. The collected data included thepatient’s demographic characteristics (age, sex, height, weight, body mass index—BMI, Haller index), treatment outcomes (duration of surgery, length of hospital stay, intraoperative complications, early and late complications, postoperative analgesia), and overall patient and legal guardian satisfaction two years after the procedure. Results: A total of 22 male and 8 female patients were included in the study. The median age was 15 years (interquartile range—IQR 14, 16), and the median BMI was 18.5 kg/m^2^ (IQR 17.7, 20.4) and 18.2 kg/m^2^ (IQR 16.9, 18.6) for males and females, respectively. The median CT Haller index was 3.67 (IQR 3.48, 4.09) for male and 3.69 (IQR 3.45, 3.9) for female patients. The median surgery duration was 120 min (IQR 100, 130), and the median hospital stay length was 8.5 days (IQR 8, 9.75). Indications for surgery were psychological (47%), followed by respiratory (30%) and combined respiratory-cardiac (20%) and respiratory-psychological disorders (3%). Early complications were observed in 18 patients (60%), and late complications were observed in 7 patients (23.3%). Intraoperative complications were not recorded. The most common early complications were pneumothorax and subcutaneous emphysema (30%), while late complications included bar displacement (10%) and deformity recurrence (6.6%). Overall, the procedure was successful in 93.1% of patients. Upon arrival home, 22 patients (81.5%) used analgesics for at least one day, up to a maximum of six months. A total of 23 (76.7%) operated patients determined that the surgical procedure had excellent results (grade 5), 4 (13.3%) patients report a good result (grade 4), 1(3.3%) patient reported a mediocre result, and one patient (3.3%) reported a bad result (grades 3 and 2, respectively). Conclusion: The Nuss procedure is a safe and effective method for treating funnel chest in children and adolescents. It also providesexcellent cosmetic and aesthetic results as well as subjective satisfaction with the outcome of surgical treatment.

## 1. Introduction

Depression of the sternum, which is also known as funnel chest or medically as pectus excavatum, is the most common chest wall deformity in children, with an incidence of 1 in 300–400 births, and may be a part of different syndromes [1,2]. It is caused by abnormal bilateral growth of the sternum and usually 4–5 rib bones [2]. The male predominance is 2–9:1, and it is usually seen in Caucasians [3]. Pectus excavatum can be an isolated abnormality and can be a part of certainsyndromes or other pathological conditions that usually involve connective tissue [1,2,3,4]. Additionally, familial inheritance has been documented, with a familial occurrence rate of 54% [5]. This condition can affect the cardiopulmonary function and can cause chest pain, dyspnoea, and exercise intolerance in some patients [6]. Besides that, the psychological aspect of shame, anxiety, depression, and distress are well known.

An important part of the standard preoperative assessment of patients who are scheduled for corrective surgery is standard radiological imaging for anatomic assessment and for the documentation of the dimensions of the chest [6]. A standard chest X-ray is used in the initial assessment of the patients because it is widelyavailable and allows the indices of severityto be measured [4,6]. Astandard chest X-ray is also helpful in patients with recurrent pectus excavatum because it shows the extent of abnormal cartilage calcification. A computed tomographic scan (CT) of the chest can provide a clearer picture of the deformity and the bony and cartilaginous skeleton in three dimensions and allows the Haller indexto be calculated [4,6].

There are several treatment options for pectus excavatum. Traditionally, an open repair (Ravitch technique) isperformed, in which the abnormal cartilage is resected, and the sternum is fractured and fixed in a corrected position [1,7,8,9,10]. Recently, the Ravitch technique has been supplanted by the significantly less invasive Nuss procedure, which has been standardized as an operative treatment of choice for most cases of pectus excavatum. A curved bar is passed upside down either blindly or with thoracoscopic assistance and is flipped into position under the sternum, effectively lifting the sternum and chest wall into a corrected position. The Nuss procedure gained popularity amongst pediatric surgeons and has been shown to have minimal blood loss, shorter operating time with good postoperative results and satisfaction, and a low rate of complications [1,7,8,9,10,11]. The proper and best age for operative treatment and for the removal of the sternal corrective bar is still controversial [12]. Corrective surgery and early bar removal in younger children may result in a higher risk of recurrence [4,12,13].

The aim of this study was to analyze efficiency with the short- and long-term outcomes of the Nuss corrective procedure for the treatment of pectus excavatum in young adolescents at our hospital.

## 2. Materials and Methods

### 2.1. Patients

The case records of 30 pediatric patients with median age of 15 years (IQR 14, 16) who had undergone a corrective Nuss procedure duepectus excavatum in the period from 1 January 2014 to 31 December 2020 at Department of Pediatric surgery, University Hospital of Split, were retrospectively reviewed. Inclusion criteria were children of both genders with pectus excavatum that had been treated with the Nuss procedure in our institution during selected study period. Exclusion criteria were patients who had been operated on usingother surgical techniques, intraoperative conversion to any other surgical technique, and patients with incomplete data and who had no possibility of providing a long-term satisfaction grade.

### 2.2. Outcomes of the Study

The primary outcome of the study was to determine the factors influencing the outcomes of treatment, whereas the secondary outcomes of the study were final treatment outcome, intraoperative and postoperative complications, rate of recurrences, the long-term grade of patient satisfaction, surgery duration, duration and type of analgesics used after surgery, and length of hospital stay.

### 2.3. Study Design

The study was designed as a retrospective observational study with a prospective arm where there was active communication with the patients and their parents or legal guardians to determine the grade of satisfaction. Six variables that could potentially affect treatment outcomes were examined to investigate which of them had an impact on the final outcome (patient satisfaction). For this purpose, the patients were divided into two groups. The first group consisted of patients who rated the final outcome as excellent or very good, and the second group consisted of those who rated the final outcome as mediocre or bad. The following parameters were analyzed for each patient included in study: age, gender, body weight and height (body mass index—BMI), indications for surgical repair, X-Ray and CT Haller indexes, duration of surgery, operative findings, use of analgesia (during the hospital stay and at home after discharge), length of hospital stay, intraoperative and postoperative complications, and grade of long-term satisfaction (1—no results, 2—bad result, 3—mediocre result, 4—good result, 5—excellent result). During the late follow-up, one patient was excluded from satisfaction analysis. The patient was treated for a severe depression prior to and after the surgery. One year after the surgery, the patient committed suicide.

The study was approved by the Ethics Review Board of University hospital of Split (reference No. 500-03/20-01/117; date of approval: 4 December 2020).

### 2.4. Anesthesia Procedure

An intravenous pathway was placed in the operating room. All patients were under general anesthesia, which was induced using fentanyl citrate (Fentanyl Piramal, West Drayton, UK) andpropofol 1% (Propofol, Fresenius Kabi Austria GmbH^©^, Linz, Austria), and muscle relaxation was achieved by vecuromiumbromide (Vecurol, Demo S.A., Thermi, Greece). After the induction of anaesthesia, tracheal intubation by endotracheal tube (Shiley™ cuffed endotracheal tubes, Covidien, Mansfield, MA, USA) was performed. Standard intraoperative monitoring included arterial blood pressure monitoring, electrocardiographic monitoring, heart rate, and peripheral oxygen saturation (Draeger-Perseus A500 Anesthesia Device Monitor, Denver, CO, USA). To measure the depth of anesthesia, a bispectral index monitoring system (BIS™ brain monitoring System, Covidien, San Jose, CA, USA) was used.

### 2.5. Surgical Technique

**Figure 1:**
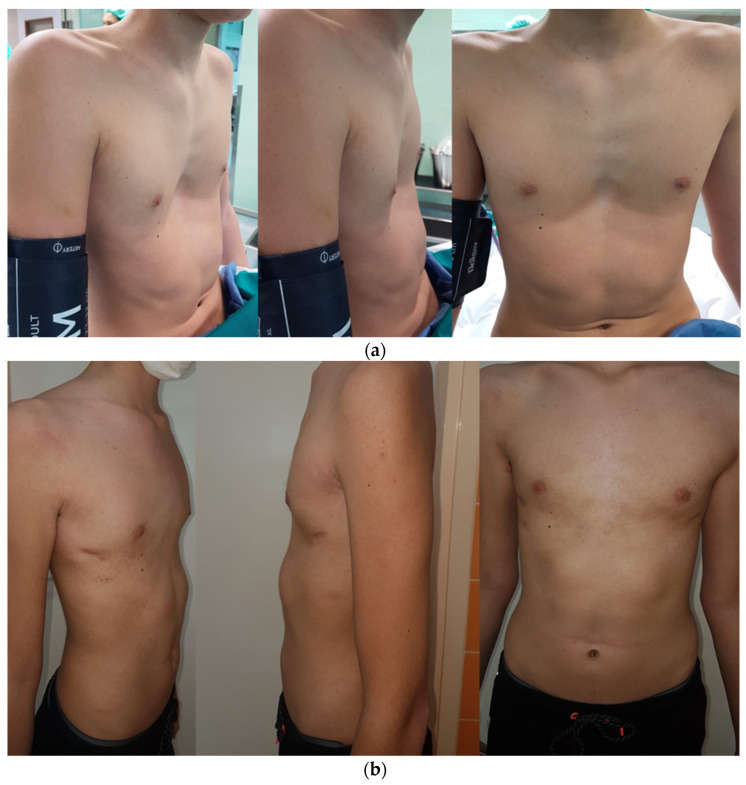
(**a**) Preoperative presentation of 16-year-old male patient with pectus excavatum scheduled for corrective Nuss procedure. (**b**) Postoperative result of the same patient two weeks after surgery.

**Figure 2:**
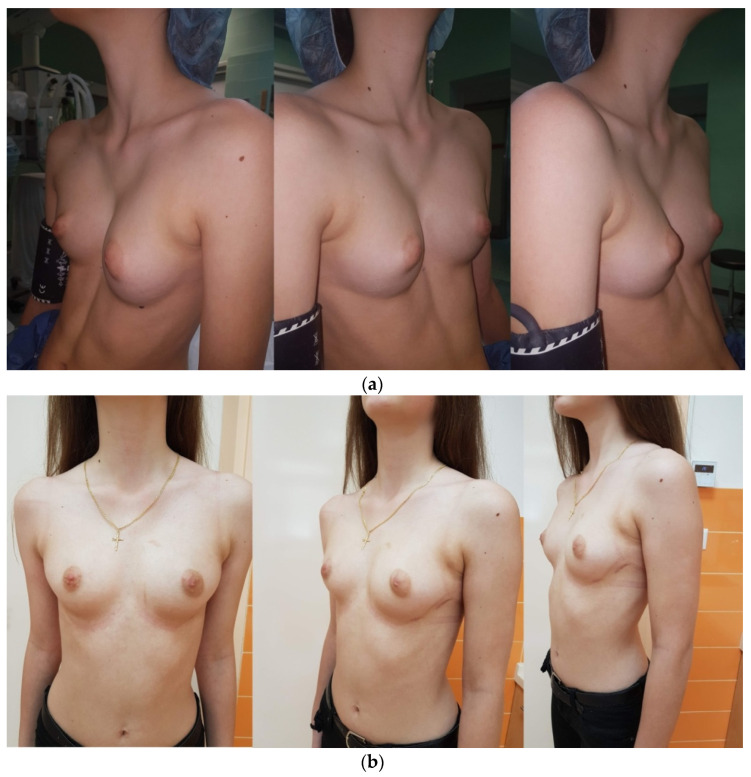
(**a**) Preoperative presentation of 15-year-old female patient with pectus excavatumscheduled for corrective Nuss procedure. (**b**) Postoperative result of the same patient two weeks after surgery.

After placing and adjusting the patient on the operating table, standard thorax measurementis performed. A corrective titanium plates (Instrumentaria d.d., Sesvete, Croatia) that are 3 mm thick, 13 mm wide, and of different lengths are premodeled according to individual measurements (260 mm–440 mm). The locationof the implantation and lateral incisions in the horizontal line of the largest recess is determined. This is followed by modeling the tile with special bending and straightening instruments (Instrumentaria d.d., Sesvete, Croatia). Two lateral incisions are made in ahorizontal line in the position of the largest indentation. Subcutaneous tissue is undermined, and space is created for the plate and stabilizers partially under the muscle and the muscular fascia as well as under the skin in the lateral part. A 5 mm thoracoscope (Olympus, Tokyo, Japan) is inserted into the right hemithorax through the intercostal space under the lateral incision, and anartificial pneumothorax is achieved with low CO_2_ pressure (2–3 mmHg). The passing of the titanium guide is controlled with a thoracoscope, which is introduced from the same side, ipsilateral to thethoracoscope. Gradually, the titanium guide is passed retrosternally, bluntly dissecting the mediastinal pleura along the pericardium to the opposite side (left) again to the projection of the largest protrusion or indentation. The titanium rod is guidedto exit at the left side of the chest through the previously formed lateral incision, and two cotton straps are tied to it, which are pushed out to the right side of the chest using the same pathway. The straps are untied from the titanium guide and are carefully tied to the correction plate on the left side, serving as a support during the pull of the plate to the left side of the chest along the formed path. After dragging and positioning the corrective bar, special rotators are used to turn the bar into its final and proper position. After achieving a satisfactory position and the achieved hypercorrection of the chest wall, the plate bar is fixed bilaterally with stabilizers/plate holders for ribs (Instrumentaria d.d., Sesvete, Croatia) that are 60 mm long and 16 mm wide. The stabilizers are secured with an M4 screws (Instrumentaria d.d., Sesvete, Croatia) in the central part and with two double polyester polyfilament non-resorptive sutures (Ethibond Excel^®^ 2-0, Ethicon, Johnson & Johnson, Cincinnati, OH, USA) through both stabilizer holes on each side for the surrounding muscles. A chest drain of 20 Fr in size (Vygon^®^, Norristown, Philadelphia, PA, USA) is placed at the site of insertion of the thoracoscope to aid in the re-expansion of the right lung. Above the correction plate outlet, the muscle is closed so that there is no leakage of air from and into the chest. Another check for residual pneumothorax and subcutaneous emphysema is performed. This is followed by the closure of the wound usinganatomical layers. Preoperative and postoperative presentation of male and female patients who underwent a corrective Nuss procedure is shown in Figure 1 and Figure 2, respectively. Bar removal is scheduled 2 to 4 years after the corrective surgery. All patients were operated on by the same surgeon (T.Š.).

### 2.6. Postoperative Protocol and Follow-Up

**Figure 3:**
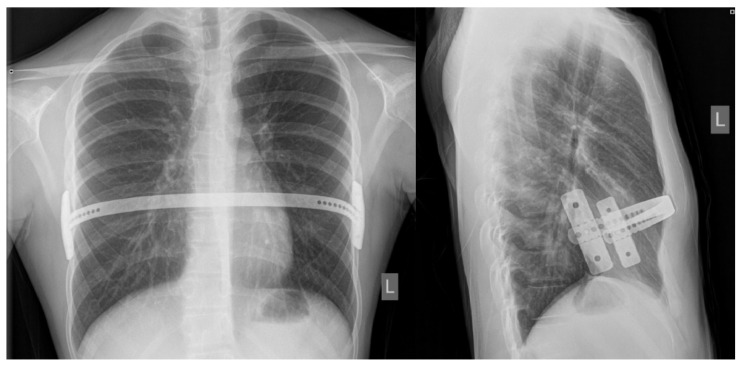
Bar position at postoperative chest X-ray: posteroanterior (**left**) and laterolateral (**right**) views.

After the surgical procedure, the patients are observed in the intensive care unit until they areoff opioid intravenous medications and after the eventual early complications are resolved. Patient-controlled analgesia, using *morphine* (Morphine hydrochloride, Alkaloid, Skopje, Republic of North Macedonia) or *fentanyl* (Fentanyl Piramal, West Drayton, UK), is started in the operating room and is gradually switched to oral pain medications over the next 2–3 days. Other strategies for controlling postoperative pain include oral non-steroidal anti-inflammatory drugs (NSAIDs): *ibuprofen* (Brufen, AbbVie, Campoverde, Italy), *metamizole sodium hydrate* (Alkagin^®^, Skopje, Republic of North Macedonia), *paracetamol* (Perfalgan, Bristol-Myers Squibb Pharmaceuticals limited, Bristol, UK),and anxiolytic/spasmolytics such as *diazepam* (Normabel, Belupo, Koprivnica, Croatia). Stool laxatives and emollients are given to prevent constipation while intravenous fluids and proton pump inhibitors are given to reduce the side effects of oral NSAIDs on the gastrointestinal tract. Pulmonary hygiene using an incentive spirometer begins a few hours after surgery and continues for several weeks to prevent lung collapse and pneumonia. Respiratory therapists are helpful in providing guidance on the proper use of the stimulus spirometer. Recovery begins on the first postoperative day with the help of a physiotherapist;on the same day, a chest X-Ray is performed to define possible early complications (pneumothorax, subcutaneous emphysema or effusions) and bar position (Figure 3). Patients are instructed to lie on their backs and to avoid any pressure on the sides of the chest during the first six weeks after surgery. Patients are discharged homeafter they are free from intravenous analgesics. At home, patients are encouraged to walk as much as possible and to do deep breathing exercises using a stimulating spirometer several times a day. Patients havefollow–up appointments at the outpatient clinic after the first, second, fourth, and sixth weeks following surgery, and any possible complications are noted. After the six and twelve month periods, another control exam is conductedat outpatient clinics as a follow-up to define the degree of pain, medication use and duration, and quality of physical therapy. Patients were screened for surgical complications according to Clavien–Dindo classification [14].

### 2.7. Statistical Analysis

The collected data were processed using the Microsoft Office software package. Data were analyzed using Microsoft Excel for Windows versions 11.0 (Microsoft Corporation, Redmond, WA, USA) and SPSS 24.0 (IBM Corp, Armonk, NY, USA). A two-sided Fisher’s exact test was used for a comparative analyses of the factors that may influence the final outcome of the study. *p* values < 0.05 were considered significant. The distribution of the quantitative data was described by median and interquartile ranges (IQR), while absolute numbers and percentages were used to describe categorical data.

## 3. Results

A total of 30 patients, 22 (73.3%) males, with median age 15 (IQR 14, 16) years, who received Nuss correction for pectus excavatum were included in the study. Most of the patients were 14 and 16 years old at the time of the procedure (*n* = 20, 66%).The median BMI was 18.5 kg/m^2^ (IQR 17.7, 20.4) and 18.2 kg/m^2^ (IQR 16.9, 18.6) for males and females, respectively. The median CT Haller index was 3.67 (IQR 3.48, 4.09) for male and 3.69 (IQR 3.45, 3.9) for female patients.

Demographic data and measurements of the operated patients are depicted in Table 1. Indications for surgical corrective treatment were as follows: psychological and anxiety disorders and psychosocial problems (*n* = 14, 46.7%), respiratory disorders (*n* = 9, 30%), cardio-respiratory disorders (*n* = 6, 20%), and a combination of respiratory and psychosocial disorders (*n* = 1, 3.3%).

The median surgery duration was 120 min (IQR 100; 130). The median length of hospitalization was 8.5 days (IQR 8; 9.75). Postoperative analgesia was used by all patients. Morphine was used in 93.1% of patients, followed by ibuprofen (86.2%) and paracetamol (75.9%). The treatment outcomes are presented in Table 2. Upon arrival home, 22 patients used analgesics for at least one day, up to a maximum of six months, while 8 of them did not use any.A total of 23 (76.7%) operated patients reported that the surgical procedure had excellent results (grade 5) o, 4 (13.3%) of them reported a good result (grade 4), one (3.3%) patient reported a mediocre result, and one (3.3%) reported a bad result (grades 3 and 2, respectively) (Table 2).The overall success of the procedure was achieved in 93.1% of the patients.

Early complications were recorded in 18 patients (60%), and late complications were reported in 7 (23.3%), while intraoperative complications were not recorded. The most common early complications were pneumothorax and emphysema, followed by pleural effusion and pneumonia, while one patient had arrhythmia, pneumomediastinum, and mild mitral regurgitation. The most common late complication was plate displacement, followed by recurrence, hypercorrection, and pericardial effusion. It was also noted that 23 patients (76.7%) were short-term subfebrile after surgery. The list of complications by patient is shown in Table 3.

All of the listed early pneumothoraces, emphysemas, and pleural effusions were treated conservatively and resolved spontaneously within two weeks. Two cases of early pneumonia were treated conservatively with antibiotics. The patient with early arrhythmia and mild mitral insufficiency that was presented even prior the surgery was followed-up with by pediatric cardiologist, and the condition resolved spontaneously without medication within one month after the surgery. Pneumomediastinum was treated conservatively and was resolved after three weeks of follow-up. All three late bar migrations were less than 40 degrees of rotatory migration (rotational bar movement-torsion) and did not need surgical reintervention. The two listed recurrences were defined as very poor result after the bar removal and the presence of pectus excavatum. One case presented with severe deformity and was operated upon using conventional surgical technique—resection and correction of costal cartilages, while other one rejected a re-do surgery due to the much smaller deformity and the significantly smaller pectus excavatum. The patients with hypercorrection were not treated in any way. The patient with late pericardial effusion was treated by a pediatric cardiologist, and the condition was resolved throughconservative treatment with NSAIDs.

Surgical complications according to Clavien–Dindo classification are shown in Table 4.

Although mediocre and bad results were only recoded in male patients, no statistically significant difference was found in regard tothe gender of the patient (*p* = 1.000) as a variable that could have potentially affected the final satisfaction of the patient. The age of the patient also did not affect the final outcome (*p* = 1.000). Although mediocre and bad results were only recorded in patients with a of height ≥ 180 cm and with a weight ≥ 70 kg, no statistically significant difference was found for these two parameters (*p* = 0.222 for both variables). BMI ≥ 24 kg/m^2^ as well as CT Haller index ≥ 5 were found as statistically significant variables thatwere more frequently associated with the patient being less satisfied with the outcome of the procedure. In all of the patients who reported mediocre and bad results, the BMI was ≥24 kg/m^2^ and the CT Haller index was ≥5 (*p* = 0.036 for both variables). These results are summarized in Table 5.

## 4. Discussion

The present study investigated the outcomes of the treatment of pectus excavatum using the minimally invasive Nuss method. The results of this study showed that the Nuss procedure is a safe and effective method for the treatment of pectus excavatum in children and adolescents. The Nuss method is related toexcellent cosmetic and aesthetic results and very good subjective satisfaction with the final outcome of the surgical procedure. The percentage of successful treatments is over 90%, and there is a very low incidence of recurrence. We also confirmed statements from the literature that the most common indication for the correction of pectus excavatum is of a psychological nature and that the anomaly is significantly more frequent in males.

Conservative treatment is considered to provide a good alternative for patients who refuse surgery and who arein a very young age group [4,13,15]. For somewhat more severe forms of pectus excavatum, surgical intervention is still a treatment of choice, and in the last three decades, minimally invasive techniques have gained a lot of popularity worldwide, especially for use in children [7,16,17]. As the best age for corrective surgery has not been precisely defined, which is also the case for minimally invasive surgical treatment as well, we have presented our data and treatment outcomes after the Nuss procedure. An operative correction in younger children (<10 years of age) has a high risk of recurrence after bar removal (again < 10 years of age) [12], but most authors imply that it is recommended to perform the procedure in children who arein the age groups of 12 to 14 years [18]. The median age of the patients in our study was 15 years, which is consistent with the above-mentioned statements.

The Haller index is defined as a ratio of the measure of the transverse diameter of the chest that is divided by the sagittal measure of the distance from the sternum to the vertebral body. The normal value of the Haller index is <2. A mild deformity is associated with values of 2–3.2; a moderate deformity is represented by values of 3.2–3.5; and a severe deformity is associated with values ≥3.5. The median Haller index in the patients who underwent surgery in literature is about 3.7, which corresponds with the results of our study [19,20]. Our study demonstrated that a high CT Haller index (≥5), as well asa BMI ≥ 24 kg/m^2^ were more frequently associated with the patient being less satisfied with the procedure, which is probably due to the patients often having a more pronounced deformity. In our study, no significant difference was found in regard to weight and height, although mediocre and poor results were reported in patients with a height ≥ 180 cm and a weight ≥ 70 kg. These parameters would also probably influence final satisfaction significantly on a larger sample size.

Indications for the surgical correction of this anomaly vary and range from moderate or severe cardio-respiratory disorders to psychological disturbances [19,20,21]. In our study, the main indication for surgical correction of pectus excavatum was psychological disturbance. Only a lower proportion of patients had respiratory problems (obstructive and restrictive disorders) or a combination of respiratory-cardiac and respiratory-psychological disorders. A similar result was recorded in comparative studies. Pawlak et al. also reported psychological disturbances (cosmetic reasons) as the most common indication for surgery, followed by respiratory problems (shortness of breath on exertion), and finally Haller index ≥3.5 [21]. Loos et al. reported exercise intolerance and psychological problems as the main indications for surgery, followed by shortness of breath at rest, angina pectoris, chest pain unrelated to angina, palpitations, and fatigue in a lower proportion of the patients [20].

In the literature, the duration of the Nuss procedure varies from 30 to 75 min [20,21,22]. The surgery duration depends on many factors, including the experience of the surgeon, the number of performed cases, and the learning curve. In our study, the median surgery duration was higher (100 min) compared to the literature, which could be attributed to a smaller number of subjects in our study, and thus the surgeon being less experienced. The average length of hospital stay also varies among published studies and ranges from 5 to 8.5 days [20,22,23]. In our study, the median length of hospital stay was 8.5 days, which is within the mentioned range but is closer to the upper limit of the range.

One of the main treatment outcomes, according to which the success of a particular surgical technique is measured, is the complication rate. Complications of corrective surgery forpectus excavatum can be divided into early and late complications. Most of the patients in our study presented some of the early complications, while late complications were noted in a significantly lower percentage of patients. Most often, these were transient, reversible complications that were not life threatening. As many as 60% of the early complications were emphysema and pneumothorax that were the result of carbon dioxide insufflation during the procedure. A low percentage of pleural effusions or pneumonia wasrecorded. The most common early complication was pneumothorax, which some authors do not even consider to be a complication [24]. In the literature, the incidence of pneumothorax varies from 4.2% to 30% [22,25,26,27], while pleural effusion has been reported in 2.5% to 10% of cases [28,29]. These findings are in line with our results. Of the late complications in our study, the most common were bar displacement (10%) and recurrence (6.7%). The displacement of the correction bar can be a serious complication resulting fromthe Nuss procedure, with a frequency ranging from 1–33% [24,25,26]. To ensure the stability of the correction bar, it is recommended to avoid the use of resorbable sutures for the fixation of the implants [23]. A similar distribution of early and late complications has been reported in other published studies [21,23]. No intraoperative complications were recorded in our study, while the incidence of intraoperative complications in comparative studies ranged from 4 to 10% [21,23,30]. Pericardial damage, heart failure as a result of pericardial injury, sternal rupture due to excessive force, and ruptures of the intercostal space are the most commonly reported complications [21,23,30,31].

The cosmetic effects after corrective surgery with the Nuss procedure are quite satisfactory. According to various studies, surgery is considered successful in more than 90% of cases [25,30]. Our study supports this finding because the deformity was successfully corrected in 93.1% of patients.

Finally, all participants from our study were contacted by telephone at least two years after the procedure, and information related to the use of analgesics after discharge and their satisfaction with the outcome of the surgery were recorded. As many as 81.5% of patients used analgesics at home, with NSAIDs being used the most often. From the total number of operated patients 76.7% of patients communicate an excellent result, while 13.3% of them rated the success as very good. In summary, we can conclude that 90% of the examined patients were satisfied with the final outcome of the surgical procedure. One patient, who had various comorbidities and psychological problems, committed suicide after surgery. High rates of satisfaction with treatment outcomes, ranging from 96.2 to 98.7% were recorded in comparative studies [21,23].

The limitations of the present study are its retrospective and single-centre design as well as its weak sample size, but the database for all of the listed patients was complete, and we were able to the contact patients in the current present time. Additional prospective, multi-center studies with a larger sample size are important to confirm our findings. The strength of this study is the long-term follow-up and evaluation of the long-term patient satisfaction.

## 5. Conclusions

The Nuss method is a safe and effective minimally invasive surgical procedure for the repair of pectus excavatum that is able to achieve good cosmetic and aesthetic results as well as excellent late subjective patient satisfaction with the final result.

## Figures and Tables

**Table 1:** Demographic data of the patients.

Data	Values
Age (years); median (IQR)	15 (14, 16)
Gender	
Male; *n* (%)	22 (73.3)
Female; *n* (%)	8 (26.7)
Height (cm)	
Male; median (IQR)	184.5 (174, 186.8)
Female; median (IQR)	173 (168.8, 174.5)
Weight (kg)	
Male; median (IQR)	65 (55, 71.8)
Female; median (IQR)	54 (50, 57.3)
BMI (kg/m^2^)	
Male; median (IQR)	18.5 (17.7, 20.4)
Female; median (IQR)	18.2 (16.9, 18.6)
CT Haller index	
Male; median (IQR)	3.67 (3.48, 4.09)
Female; median (IQR)	3.69 (3.45, 3.9)

IQR—interquartile range; BMI—body mass index; CT—computed tomography.

**Table 2:** Treatment outcomes.

Outcome Value	
Operating duration (min); median (IQR)	120 (100, 130)
Hospitalization length (days); median (IQR)	8.5 (8, 9.75)
Complications; *n* (%)	
Intraoperative	0 (0)
Early	18 (60%)
Late	7 (23.3%)
Analgesia; *n* (%)	
Postoperative in hospital	30 (100%)
After discharge	22 (81.5%)
Satisfactory grade (1–5); *n* (%)	
Excellent (5)	23 (76.7%)
Very good (4)	4 (13.3%)
Mediocre (3)	1(3.3%)
Bad result (2)	1(3.3%)
No result (1)	0 (0)

IQR—interquartile range.

**Table 3:** Early and late postoperative complications after Nuss procedure.

**Early Complications**	***n* (%)**
Pneumothorax and/or emphysema	9 (30%)
Pleural effusion	4 (13.3%)
Pneumonia	2 (6.6%)
Arrhythmia	1 (3.3%)
Pneumomediastinum	1 (3.3%)
Mild mitral insufficiency	1 (3.3%)
**Late Complications**	***n* (%)**
Bar migration	3 (10%)
Recurrence	2 (6.6%)
Hypercorrection	1 (3.3%)
Pericardial effusion	1 (3.3%)

**Table 4:** Clavien–Dindo classification of surgical complications.

Grade, *n* (%)	Group I	Group II	Total
	Early Postoperative Complications	Late Postoperative Complications	
I	17 (56.6)	5 (16.6)	22 (73.3)
II	1 (3.3)	1 (3.3)	2 (6.6)
III a	0 (0)	0 (0)	0 (0)
III b	0 (0)	1 (3.3)	1 (3.3)
IV	0 (0)	0 (0)	0 (0)
V	0 (0)	0 (0)	0 (0)

**Table 5:** Factors influencing the outcomes of treatment after Nuss corrective surgery.

Variable		Outcome		*p* *
		Excellent or Very Good	Mediocre or Bad	
Gender	Male	19	2	1.000
	Female	8	0	
Age (years)	<16	17	1	1.000
	≥16	10	1	
Height (cm)	<180	14	0	0.222
	≥180	11	2	
Weight (kg)	<70	14	0	0.222
	≥70	11	2	
BMI (kg/m^2^)	<24	23	0	0.036
	≥24	4	2	
CT Haller index	<5.0	23	0	0.036
	≥5.0	4	2	

* Fisher’s exact test; BMI—body mass index; CT—computed tomography.

## Data Availability

The data presented in this study are available upon request from the corresponding author. Due to the protection of personal data, the data are not publicly available.
